# Changes in peripheral AD biomarkers in a randomized controlled trial of Yoga vs. memory training in older women at risk for Alzheimer’s disease

**DOI:** 10.1002/alz.086035

**Published:** 2025-01-09

**Authors:** Helen Lavretsky

**Affiliations:** ^1^ UCLA, Los Angeles, CA USA

## Abstract

**Background:**

Biomarkers such as amyloid beta (Aβ) peptides and tau protein can correlate with changes in cognition and mood symptoms associated with AD. We aimed to investigate the blood biomarker response to a Kundalini Yoga (KY) compared to Memory Enhancement Training (MET) intervention as well as determine the predictive value of blood biomarkers on outcomes in a randomized trial targeting women at risk for Alzheimer's disease.

**Method:**

Women aged 60 years old and older with subjective memory complaints and high cardiovascular risk score were randomized to a 12‐week RCT of Kundalini yoga or Memory enhancement training (MET). We obtained blood samples at baseline and week 24 (6 months) to measures AB40; AB42 and p‐Tau. Participants completed the Beck Depression Inventory (BDI) for depressive symptoms, and Memory Functioning Questionnaire (MFQ) for subjective memory reports. Regression models were used to examine whether baseline and changes in biomarker levels including Aβ40, Aβ42, Aβ42/40 ratio and tau were associated with changes in outcomes at 24 weeks.

**Result:**

A total of 79 patients (KY=40; MET=39) were randomized, and 63 completed the 24‐week follow‐up. There were no group differences in AD biomarkers at baseline or at 24‐week follow‐up. Baseline levels of Aβ40 were significantly associated with an increase in subjective memory (MFQ Frequency of Forgetting; r = 0.27, p=0.04). An increase in Aβ40 levels was significantly associated with a decrease in BDI (r = ‐0.33, p=0.02). There was no significant association of changes in Aβ42, Aβ42/40 ratio and tau with any outcomes.

**Conclusion:**

Our findings indicate that greater levels of Aβ40 at baseline are associated with an increase in subjective memory at follow‐up and an increase in Aβ40 levels is correlated to a decrease in depressive symptoms as expected. It has been established that a low ratio of Aβ42/Aβ40 may reflect selective depletion of Aβ42 due to deposition in the growing plaques and subsequent AD pathology, while Aβ40 may have opposing effects on amyloid deposition and be neuroprotective. Our findings support the evidence that yoga can reduce stress, depression, and anxiety and may have an impact on peripheral AD biomarkers.

